# Collateral projections of neurons in laminae I, III, and IV of rat spinal cord to thalamus, periaqueductal gray matter, and lateral parabrachial area

**DOI:** 10.1002/cne.22081

**Published:** 2009-04-03

**Authors:** Khulood M Al-Khater, Andrew J Todd

**Affiliations:** Neuroscience and Molecular Pharmacology, Faculty of Biomedical and Life Sciences, University of GlasgowGlasgow, G12 8QQ United Kingdom

**Keywords:** neurokinin 1 receptor, dorsal horn, spinothalamic tract, spinomesencephalic tract, confocal microscopy

## Abstract

Projection neurons in lamina I, together with those in laminae III–IV that express the neurokinin 1 receptor (NK1r), form a major route through which nociceptive information reaches the brain. Axons of these cells innervate various targets, including thalamus, periaqueductal gray matter (PAG), and lateral parabrachial area (LPb), and many cells project to more than one target. The aims of this study were to quantify projections from cervical enlargement to PAG and LPb, to determine the proportion of spinothalamic neurons at lumbar and cervical levels that were labelled from PAG and LPb, and to investigate morphological differences between projection populations. The C7 segment contained fewer lamina I spinoparabrachial cells than L4, but a similar number of spino-PAG cells. Virtually all spinothalamic lamina I neurons at both levels were labelled from LPb and between one-third and one-half from PAG. This suggests that significant numbers project to all three targets. Spinothalamic lamina I neurons differed from those labelled only from LPb in that they were generally larger, were more often multipolar, and (in cervical enlargement) had stronger NK1r immunoreactivity. Most lamina III/IV NK1r cells at both levels projected to LPb, but few were labelled from PAG. The great majority of these cells in C7 and over one-fourth of those in L4 were spinothalamic, and at each level some projected to both thalamus and LPb. These results confirm that neurons in these laminae have extensive collateral projections and suggest that different neuronal subpopulations in lamina I have characteristic patterns of supraspinal projection. J. Comp. Neurol. 515:629–646, 2009.

The superficial dorsal horn of the spinal cord (laminae I and II; Rexed, 1952) receives a major input from nociceptive primary afferents, many of which contain substance P. This acts on the neurokinin 1 receptor (NK1r), which is found on a significant proportion of neurons in lamina I as well as on some of those in laminae III–VI (Bleazard et al., 1994; Liu et al., 1994; Nakaya et al., 1994; Brown et al., 1995; Littlewood et al., 1995). In the dorsal horn, the NK1r is thought to be restricted to cells that respond to noxious stimuli (Henry, 1976; Salter and Henry, 1991). Lamina I contains numerous projection neurons, most of which express the NK1r (Ding et al., 1995; Marshall et al., 1996; Li et al., 1998; Todd et al., 2000; Spike et al., 2003). There is also a population of large NK1r-immunoreactive projection neurons in laminae III and IV with prominent dorsal dendrites that enter lamina I (Marshall et al., 1996; Naim et al., 1997; Todd et al., 2000). Cells belonging to both of these populations send predominantly contralateral projections to several brain regions, including the lateral parabrachial area (LPb), periaqueductal gray matter (PAG), and thalamus.

The spinothalamic tract is an important pathway, providing direct spinal input to the thalamus, from which information is transmitted to cortical areas that underlie pain perception. However, we recently reported that there are only ∼15 lamina I spinothalamic neurons per side in the L4 segment of the rat (Al-Khater et al., 2008). Because there are around 400 lamina I projection neurons on each side in this segment (Spike et al., 2003), the proportion projecting to thalamus is only 4%. In contrast, spinothalamic lamina I neurons were much more numerous in the cervical enlargement (∼90 cells per side in C7). However, the total number of projection neurons in this lamina in C7 is not known, so we are unable to estimate the proportion belonging to the spinothalamic tract. The contribution to this tract from lamina III/IV NK1r projection neurons is also considerably greater in the cervical enlargement (Al-Khater et al., 2008).

Projections to LPb and PAG from lamina I in the rat lumbar enlargement are more substantial. It has been estimated that there are ∼350 spinoparabrachial neurons and 115 spino-PAG neurons on each side in this lamina in L4 (Spike et al., 2003). In addition, 66% of the large lamina III/IV NK1r cells in L4 project to LPb, although few of these cells (6%) were labelled from PAG (Todd et al., 2000). Many neurons project to more than one supraspinal target; for example, we observed that 97% of lamina I spino-PAG neurons in L4 were also labelled from LPb (Spike et al., 2003). Hylden et al. (1989) reported that >80% of spinothalamic lamina I neurons in both enlargements projected to LPb and that 30–35% of spinoparabrachial neurons were also spinothalamic. However, this is difficult to reconcile with our finding that L4 contains approximately 20 times as many spinoparabrachial as spinothalamic neurons in lamina I (Spike et al., 2003; Al-Khater et al., 2008) and with reports that there are significant differences in physiological properties between spinoparabrachial and spinothalamic lamina I neurons in the rat (Bester et al., 2000; Zhang et al., 2006). There is also evidence that some spinothalamic lamina I neurons in rat (Liu, 1986; Harmann et al., 1988) and monkey (Zhang et al., 1990) send axon collaterals to PAG, although from these studies the extent of this collateral projection appears to be very limited. In addition, it has not yet been established whether significant numbers of cells project to all three of these targets.

Lamina I projection neurons in rat, cat, and monkey can be grouped into three morphological classes: fusiform, pyramidal, and multipolar/flattened (Lima et al., 1991; Zhang et al., 1996; Zhang and Craig, 1997), and it has been suggested that these represent different functional classes (Han et al., 1998). We have previously reported that spinoparabrachial neurons belonging to each class were found with approximately equal frequency in rat lumbar cord (Spike et al., 2003). However, there is disagreement about the morphology of lamina I spinothalamic neurons in the rat (Lima and Coimbra, 1988; Yu et al., 2005). We have also identified a distinctive population of large multipolar lamina I spinoparabrachial neurons that are characterized by their high density of inhibitory and excitatory synapses and can be identified by the presence of numerous gephyrin puncta on their somata and dendrites (Puskár et al., 2001; Polgár et al., 2008). Although there are only ∼10 of these large gephyrin-coated cells on either side in L4 (corresponding to 3% of the spinoparabrachial population in this lamina), we have recently shown that they constitute ∼20% of the lamina I spinothalamic neurons at this level (Polgár et al., 2008).

In the present study, we have carried out a quantitative analysis to address a number of unresolved issues concerning projections from laminae I, III, and IV to the thalamus, LPb, and PAG. The aims were 1) to estimate the numbers of neurons that project from cervical enlargement to LPb and PAG, 2) to reassess the extent to which spinothalamic neurons can be retrogradely labelled from LPb and determine whether spinoparabrachial neurons that project to thalamus differ morphologically from those that do not, 3) to quantify collateral projections from spinothalamic neurons to the PAG, and 4) to establish whether any neurons project to all three of these targets.

## MATERIALS AND METHODS

### Surgery

All experiments were approved by the Ethical Review Process Applications Panel of the University of Glasgow and were performed in accordance with the U.K. Animals (Scientific Procedures) Act, 1986. Tissue from seven adult male Wistar rats (260–335 g; Harlan, Loughborough, United Kingdom) was used in this study. Three of these rats (experiments Pb1–3) had received two injections (100 nl each) of 4% Fluorogold (Fluorochrome Inc., Englewood, CO) into the caudal half of the thalamus, including the posterior triangular nucleus of the thalamus (PoT), together with an injection of 200 nl 1% CTb (Sigma, Poole, United Kingdom) into the LPb (both on the left side). These three rats had been used in a previous study to investigate the proportion of lamina I spinothalamic neurons in the L5 segment that belonged to the large gephyrin-coated population (Polgár et al., 2008).

The other four rats (experiments PAG1–4) were anesthetized with isofluorane and placed in a stereotaxic frame, after which anesthetic was administered through a mask attached to the frame. Each of these rats received two injections of 100 nl 4% Fluorogold targeted on the caudal thalamus on the left (as described above) and 100 nl 1% CTb (Sigma) into the PAG on the left side. Tissue from two of these animals (PAG1–2) had also been used in the study of gephyrin-coated lamina I spinothalamic neurons in L5 (as described above). The PoT nucleus was targeted for the thalamic injections, because this is a major termination zone for projections from the superficial dorsal horn (Gauriau and Bernard, 2004a), and we have shown that injections of tracer into this region label virtually all spinothalamic neurons in lamina I, together with those in laminae III and IV that express the NK1r (Al-Khater et al., 2008). All injections were made through glass micropipettes, which were left in place for 5 minutes after the completion of each injection to minimize leakage of tracer back up the track. In all cases, a different pipette was used for each tracer. The animals made an uneventful recovery from anesthesia. After a survival period of 3 days, they were reanesthetized with pentobarbitone (300 mg i.p.) and perfused through the heart with a fixative that contained 4% freshly depolymerized formaldehyde. Lumbar and cervical spinal cord segments were removed and stored in fixative for 24 hours, and the brain was cryoprotected in 30% sucrose in fixative overnight.

### Tissue processing and immunocytochemistry

Brain regions containing injection sites were cut into 100-μm-thick coronal sections with a freezing microtome. Sections that included the thalamic injection site were mounted with antifade medium and viewed with epifluorescent illumination and an UV filter set. Those through LPb or PAG were reacted with goat anti-CTb (List Biological Laboratories, Campbell, CA; diluted 1:50,000) using an immunoperoxidase method as described previously (Todd et al., 2000). In all cases, the spread of tracer from the injection sites was plotted onto drawings of the thalamus and brainstem (Paxinos and Watson, 2005), and representative examples were photographed.

The C7 and L4 spinal cord segments from all animals were initially notched on the left so that the two sides could subsequently be distinguished and were then cut into 60-μm-thick transverse sections with a Vibratome. The sections were incubated free-floating at 4°C for 3 days in guinea pig anti-Fluorogold (Protos Biotech Corp., New York, NY; 1:500), goat anti-CTb (1:5,000), and rabbit anti-NK1r (Sigma-Aldrich; 1:10,000) and then overnight in species-specific secondary antibodies that were raised in donkey and conjugated either to Alexa 488 (Invitrogen, Paisley, United Kingdom; 1:500) or to rhodamine red or Cy5 (Jackson Immunoresearch, West Grove, PA; 1:100). The sections were mounted in serial order in antifade medium and stored at −20°C.

The C6 and L5 segments from all seven rats and the C8 and L3 segments from experiments Pb1–3 were also notched on the left side and were cut into 60-μm-thick horizontal sections. Those from C8 and L3 were processed in the same way as described above, whereas sections from C6 and L5 were incubated for 3 days with guinea pig anti-Fluorogold, goat anti-CTb and mouse monoclonal antibody against gephyrin (mAb 7a; Synaptic Systems, Göttingen, Germany; 1:1,000) and then overnight in fluorescent secondary antibodies (as described above). Sections were mounted and stored at −20°C.

### Antibody characterization

The NK1r antibody (catalog No. S8305) was raised in rabbit against a peptide corresponding to amino acids 393–407 at the C-terminus of the rat NK1r, which was conjugated to keyhole limpet hemocyanin. The antibody recognises a 46-kDa band in Western blots of rat brain extracts, and this staining is specifically abolished by preabsorption of the antibody with the immunizing peptide (manufacturer's specification). It has been shown that there is no immunostaining with this antibody in sections of medulla and cervical spinal cord from mice in which the NK1r has been deleted (NK1^−/−^), whereas staining is present in sections from wild-type mice (Ptak et al., 2002).

The mouse monoclonal antibody against gephyrin was generated against an extract of rat spinal cord synaptic membranes (Pfeiffer et al., 1984) and has been extensively characterized and shown with Western blots to bind to a 93-kDa peripheral membrane protein (gephyrin) in extracts of rat brain membranes (Becker et al., 1989; Kirsch and Betz, 1993).

Goat (catalog No. 703) and guinea pig (catalog No. NM101) polyclonal antibodies were raised against CTb and Fluorogold, respectively. Specificity of each of these antibodies was shown by the lack of staining in regions of the CNS that did not contain neurons that had taken up and transported the tracer and by the presence of immunostaining in populations of neurons that are known to project to the injection sites. The specificity of the Fluorogold antibody was also directly confirmed by comparing Fluorogold fluorescence (observed with an UV filter set) with that for anti-Fluorogold in individual neurons. In all cases examined, there was a perfect match between the two types of fluorescence.

### Confocal microscopy and analysis

All analysis of lamina I neurons and of the large NK1r-immunoreactive cells in laminae III and IV was performed on the right (contralateral) dorsal horn. Transverse sections from C7 and L4 segments of all seven rats were used to analyze the numbers of retrogradely labelled lamina I neurons that contained one or both tracers. In each case, 10 or 20 sections (an alternate series) were scanned sequentially (to avoid fluorescent bleed-through) with a confocal microscope (Bio-Rad Radiance 2100; Bio-Rad, Hemel Hempstead, United Kingdom) through dry (×20) and oil-immersion (×40) lenses. Darkfield microscopy was used to distinguish laminar boundaries, and retrogradely labelled cells were judged to be in lamina I if they were very close to the dorsal border of the dorsal horn or lay dorsal to the dark band identified as lamina II with darkfield microscopy. To correct for the overcounting that results from the presence of transected cells at the section surfaces, cells were only included in the sample if their nucleus (identified as a filling defect) was entirely contained within the Vibratome section or if part of the nucleus was present in the first optical section in the z-series (corresponding to the top of the Vibratome section); they were excluded if part of the nucleus was present in the last optical section (Spike et al., 2003; Al-Khater et al., 2008). Spinothalamic lamina I neurons are infrequent in the lumbar enlargement (Al-Khater et al., 2008), so we examined 20 sections through the L4 segment to count these cells (and to determine whether they were double-labelled). We also used 20 sections to quantify spinal neurons labelled from PAG in the L4 segment, whereas 10 sections were used for the other parts of this analysis. In this way, the mean number of retrogradely labelled lamina I cells containing CTb, Fluorogold, or both tracers per 600 μm (C7 for each injection site, L4 for LPb injections) or 1,200 μm (L4 for thalamic and PAG injections) was determined for each experiment. The presence or absence of NK1r was also noted for each retrogradely labelled lamina I cell. To compare the mediolateral distribution of spinothalamic and spinoparabrachial neurons in lamina I, the locations of single- or double-labelled neurons in the sections analyzed were plotted onto an outline drawing of the dorsal horn with Neurolucida for Confocal software (MicroBrightField Inc., Colchester, VT). Lamina I was divided into three equal parts (medial, middle, and lateral), and the numbers of spinoparabrachial and spinothalamic neurons in each part were counted. For this analysis, spinothalamic neurons in the L4 segment were analyzed on 20 sections, whereas those in C7, together with spinoparabrachial neurons in both segments, were analyzed on 10 sections.

In all seven animals, the complete series of sections from C7 and L4 were also used to count the number of NK1r-immunoreactive neurons that had cell bodies in laminae III or IV and dorsal dendrites that could be followed into laminae I or II and to determine the proportion of these cells that was retrogradely labelled with one or both tracers. Darkfield microscopy was used to ensure that all cells had their somata ventral to lamina II. Sections were initially viewed with epifluorescence through a ×20 lens to identify NK1r-immunoreactive cell bodies in laminae III or IV. In most cases, it was possible to determine with epifluorescence microscopy whether the dendrites of these cells entered the superficial dorsal horn (laminae I or II). However, in some cases (particularly when dendrites had to be followed into serial sections), it was necessary to scan with the confocal microscope. This was also used to determine whether the cells were retrogradely labelled with CTb and/or Fluorogold, to measure the distance between the cell body and the overlying dorsal white matter, and to define the cells' mediolateral location. We have previously reported that, in the lumbar enlargement, the proportion of these cells retrogradely labelled from the thalamus was higher for those that were medially located. For the L4 segments, we therefore plotted the locations of all of these cells with Neurolucida for Confocal onto an outline of the dorsal horn, drew a vertical line midway through the mediolateral extent of lamina III, and divided the cells into two groups, those in the medial and those in the lateral halves of the dorsal horn.

For experiments Pb1–3, all of the horizontal sections through the C8 and L3 segments that contained retrogradely labelled lamina I neurons were scanned sequentially through dry (×20) and oil-immersion (×40) lenses to reveal NK1r, CTb, and Fluorogold. These scans were used to compare the morphology and NK1r expression of neurons that were retrogradely labelled from both thalamus and LPb with those of neurons labelled only from LPb. For C8, we obtained confocal image stacks (1 μm z-separation) through cell bodies and dendritic trees of all retrogradely labelled neurons. A much lower number of Fluorogold-labelled cells was present in L3. Therefore, in this segment, only regions that contained Fluorogold-labelled cells were scanned, but all of the retrogradely labelled cells in these scans were analyzed. Cells were excluded from the sample if they were so close to one surface of the Vibratome section that substantial parts of their proximal dendrites (and/or cell bodies) were not present in the section, because this made it impossible to allocate them to one of the three morphological classes. Drawings of the cell bodies and proximal dendrites of all of the retrogradely labelled neurons included in the sample were made with Neurolucida for Confocal software, and the presence or absence of CTb and Fluorogold was recorded for each cell. The drawings were used to analyze neuronal morphology. For each cell in the sample, morphology was assessed independently by two observers who were blind to the projection target(s), and cells were provisionally allocated to one of the following classes: fusiform, multipolar, or pyramidal (Zhang et al., 1996; Zhang and Craig, 1997). In cases of initial disagreement, the cells were reexamined and, where possible, allocated to one of these classes. A small number of cells could not be assigned to one of the three groups because they showed features that were transitional between two of the three morphological classes, and a few could not be allocated to any of these classes because of their atypical appearance (Zhang et al., 1996; Zhang and Craig, 1997). These cells were defined as “unclassified.” The maximal cross-sectional area of the soma of all cells was measured from projected confocal images with Neurolucida for Confocal (Puskár et al., 2001; Polgár et al., 2002). Strength of NK1r immunoreactivity on the plasma membrane was also recorded; because of the variation in staining intensity at different depths of the Vibratome section, a scoring system was used (Spike et al., 2003), and each cell was assigned a score ranging from 4 (strong) to 1 (very weak) or 0 (negative). Because the sample of spinothalamic neurons in the L3 segments was small (a total of 21 cells in the 3 Pb experiments), we also analyzed morphology and soma size of spinothalamic neurons from the L5 segments of these experiments. To allow unbiased analysis of morphology, the spinothalamic cells in this segment, together with a sample of cells labelled only from LPb, were drawn and assessed by two observers blind to their projection targets, as described above. However, only the spinothalamic cells from L5 were included in the analysis.

For all seven rats, horizontal sections through the C6 and L5 segments were examined with an oil-immersion (×40) lens to allow identification of the large gephyrin-coated lamina I cells, and these were then scanned sequentially to reveal gephyrin, CTb, and Fluorogold. These scans were used to determine the proportion of the gephyrin-coated cells in the C6 segment that was retrogradely labelled from thalamus, LPb, or PAG and the proportion of cells of this type in L5 that were labelled from PAG. Figures were composed with Adobe Photoshop (version 7.0). In some cases, image brightness and contrast were adjusted by using the Levels setting.

## RESULTS

### Injection sites

**Figure 1:**
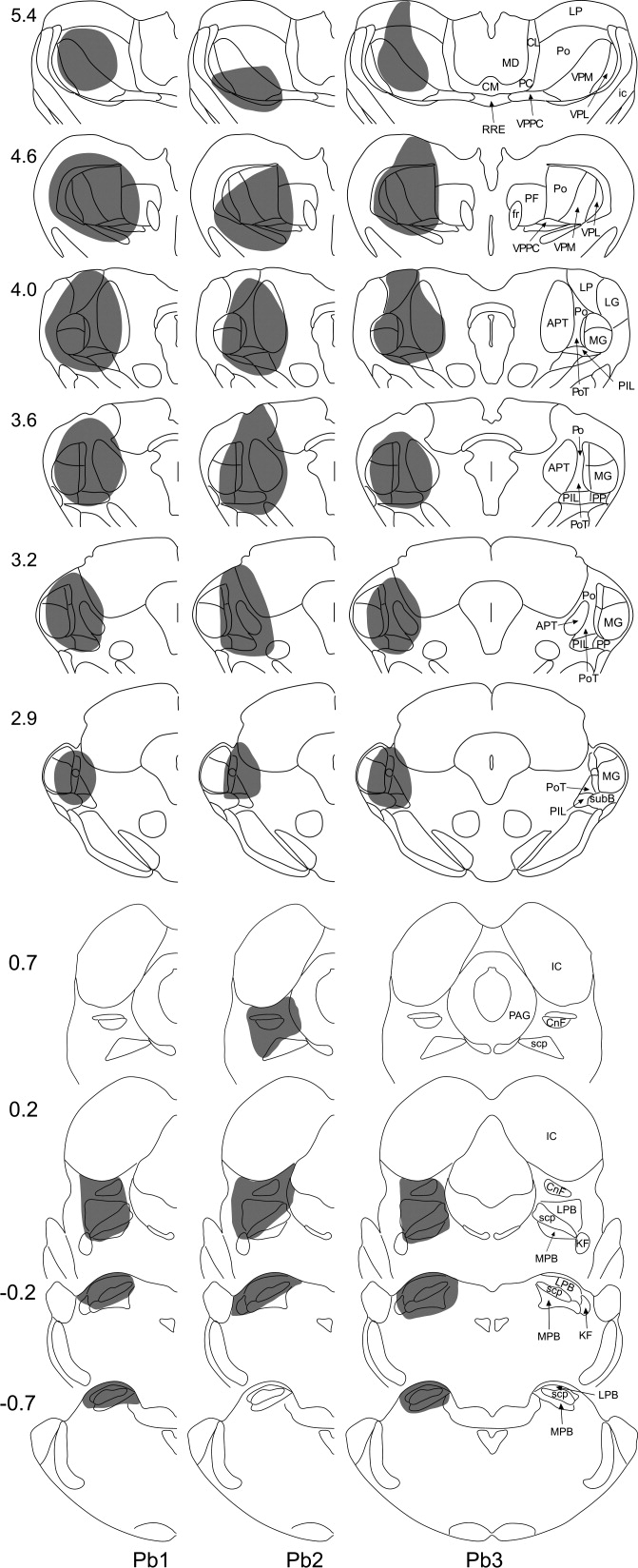
Fluorogold and CTb injection sites in experiments Pb1–3. Drawings to show the spread of tracer (shaded area) in these experiments. Each vertical column represents a single experiment, and the experiment number is shown at the bottom of the column. Numbers to the left give the approximate position of the section relative to the interaural plane. Drawings are based on those in Paxinos and Watson (2005). The upper six outlines in each column represent the Fluorogold injection (targetted on caudal thalamus), while the lower four show the spread of CTb (targetted on LPb). APT, anterior pretectal nucleus; CL, centrolateral thalamic nucleus; CM, central medial thalamic nucleus; CnF, cuneiform nucleus; fr, fasciculus retroflexus; IC, inferior colliculus; ic, internal capsule; KF, Kölliker-Fuse nucleus; LG, lateral geniculate nucleus; LP, lateral posterior thalamic nucleus; LPB, lateral parabrachial nucleus; MD, mediodorsal thalamic nucleus; MG, medial geniculate nucleus; MPB, medial parabrachial nucleus; PAG, periaqueductal gray matter; PC, paracentral thalamic nucleus; PF, parafascicular thalamic nucleus; PIL, posterior intralaminar thalamic nucleus; Po, posterior thalamic nuclear group; PoT, posterior thalamic nuclear group, triangular part; PP, peripeduncular nucleus; RRE, retrouniens area; SC, superior colliculus; scp, superior cerebellar peduncle; SubB, subbrachial nucleus; VPL, ventral posterolateral thalamic nucleus; VPM, ventral posteromedial thalamic nucleus; VPPC, ventral posterior thalamic nucleus, parvicellular part.

The spread of tracers in experiments Pb1–3 was briefly reported in our previous paper (Polgár et al., 2008), and photomicrographs of the injection sites from one case were illustrated. The drawings in Figure 1 show the spread of Fluorogold and CTb in a series of coronal sections from these experiments. These indicate that the PoT was completely filled with Fluorogold in each case, with spread into surrounding areas (other thalamic, anterior pretectal, and deep mesencephalic nuclei) but not into hypothalamus, PAG, or LPb. CTb injections filled the LPb with variable spread into medial parabrachial, cuneiform, and Kölliker-Fuse nuclei. In one case (Pb2), there was spread into the extreme caudal part of the ventrolateral PAG.

**Figure 2:**
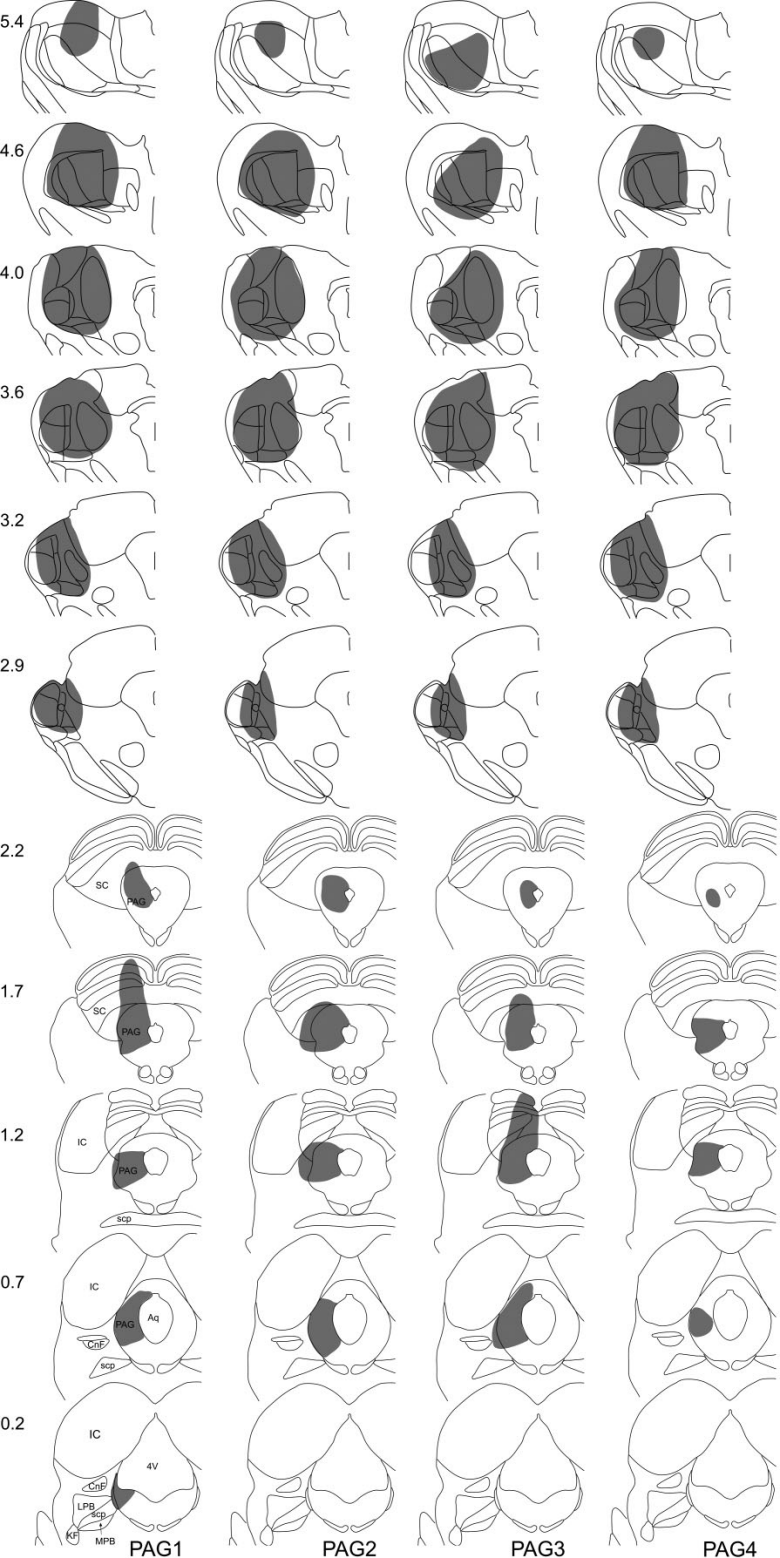
Fluorogold and CTb injection sites in experiments PAG1–4. Drawings to show the spread of tracer (shaded area) in these experiments. Each vertical column represents a single experiment, and the experiment number is shown at the bottom of the column. Numbers to the left give the approximate position of the section anterior to the interaural plane. Drawings are based on those in Paxinos and Watson (2005). The upper six outlines in each column represent the Fluorogold injection (targetted on caudal thalamus), whereas the lower five show the spread of CTb (targetted on PAG). 4V, fourth ventricle; Aq, aqueduct; SC, superior colliculus. Other abbreviations as in Figure 1.

**Figure 3:**
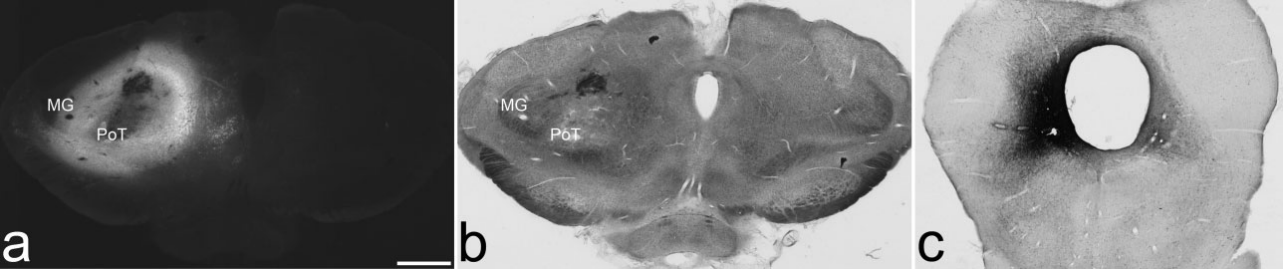
Examples of Fluorogold and CTb injection sites. **a**,**b**: Fluorescent and brightfield photomicrographs of a section (interaural ∼4 mm) through the thalamic injection site in PAG3. **c**: Section (interaural ∼0.9 mm) through the CTb injection in PAG4. The section was reacted with an immunoperoxidase method to reveal CTb. Scale bar = 1 mm.

The extent of spread of Fluorogold in experiments PAG1–4 (Fig. 2) was similar to that described above. The CTb in these experiments spread between interaural 0.4 and 2.4 mm and was largely contained within the PAG, except in PAG1 and PAG3, in which there was some spread dorsally into the superior colliculus (Fig. 2). The dorsolateral, lateral, and ventrolateral columns of the PAG were filled to a variable extent in these experiments. Examples of injection sites from the PAG experiments are shown in Figure 3.

### Analysis of lamina I projection neurons in transverse sections

This part of the study was performed on sections from the C7 and L4 segments. Neurons that were retrogradely labelled from each injection site (Fluorogold-positive from thalamus and CTb-positive from LPb or PAG) were found in lamina I and throughout the deeper laminae (III–VIII, X). The distributions of retrogradely labelled cells were generally similar to those reported for each of these targets in previous studies in the rat (Giesler et al., 1979; Menétrey et al., 1982; Cechetto et al., 1985; Liu, 1986; Harmann et al., 1988; Lima and Coimbra, 1988,^1989^; Hylden et al., 1989; Burstein et al., 1990a; Ding et al., 1995; Li et al., 1996,^1998^; Marshall et al., 1996; Keay et al., 1997; Kobayashi, 1998; Todd et al., 2000; Spike et al., 2003).

Quantitative data for retrogradely labelled lamina I cells for experiments Pb1–3 and PAG1–4 are shown in Tables 1 and 2. When results from all seven experiments were pooled, the mean numbers of neurons retrogradely labelled from the thalamus were 24.3 per 600 μm for C7 (corresponding to 91 cells in the segment) and 9.3 per 1,200 μm for L4 (corresponding to 20 cells in the segment; data from Tables 1 and 2). In experiments Pb1–3, the numbers of lamina I neurons labelled from LPb were considerably higher in L4 (80.3 cells/600 μm, corresponding to 335 in the segment) than in C7 (46 cells/600 μm, corresponding to 176 cells in the segment). In PAG1–4, the numbers of lamina I spino-PAG cells were similar in each segment (21.8/600 μm, corresponding to 83 cells in C7; 41.3/1,200 μm, corresponding to 86 cells in L4).

**TABLE 1:** Quantitative Results for Lamina I Neurons in Pb Experiments^1^

	C7					L4				
Experiment	SPb cells (600 μm)	ST cells (600 μm)	DL cells (600 μm)	DL as percentage of SPb	DL as percentage of ST	SPb cells (600 μm)	ST cells (1,200 μm)	DL cells (1,200 μm)	DL as percentage of SPb	DL as percentage of ST
Pb1	46	26	26	56.5	100	78	13	13	5.1	100
Pb2	42	18	18	42.9	100	75	8	7	8	87.5
Pb3	50	17	17	34	100	88	10	9	3.4	90
Mean	46	20.3	20.3	44.5	100	80.3	10.3	9.7	5.5	92.5

1 Counts of retrogradely labelled cells in the C7 and L4 segments from experiments in which Fluorogold was injected into thalamus and CTb into LPb. Cells labelled with Fluorogold were classified as spinothalamic (ST), those labelled with CTb as spinoparabrachial (SPb). Cells that contained both tracers are shown as double labelled (DL). In each experiment, cells were counted in 10 (SPb, ST, DL in C7, SPb in L4) or 20 (ST, DL in L4) alternate 60-μm sections. Note that, in the L4 segment, the percentage of SPb cells that were DL was determined for 10 sections, whereas the percentage of ST cells that were DL was determined for 20 sections.

**TABLE 2:** Quantitative Results for Lamina I Neurons in PAG Experiments^1^

	C7					L4				
Experiment	SPAG cells (600 μm)	ST cells (600 μm)	DL cells (600 μm)	DL as percentage of SPAG	DL as percentage of ST	SPAG cells (1,200 μm)	ST cells (1,200 μm)	DL cells (1,200 μm)	DL as percentage of SPAG	DL as percentage of ST
PAG1	25	20	13	52	65	46	12	8	17.4	66.7
PAG2	20	29	14	70	48.3	29	5	1	3.4	20
PAG3	20	29	8	40	27.6	51	15	7	13.7	46.7
PAG4	22	31	15	68.2	48.4	39	2	0	0	0
Mean	21.8	27.3	12.5	57.5	47.3	41.3	8.5	4	8.6	33.3

1 Counts of retrogradely labelled cells in the C7 and L4 segments from experiments in which Fluorogold was injected into thalamus and CTb into PAG. Cells labelled with Fluorogold were classified as spinothalamic (ST), those labelled with CTb as spino-PAG (SPAG). Cells that contained both tracers are shown as double labelled (DL). In each experiment, cells were counted in 10 (C7) or 20 (L4) alternate 60-μm sections.

**Figure 4:**
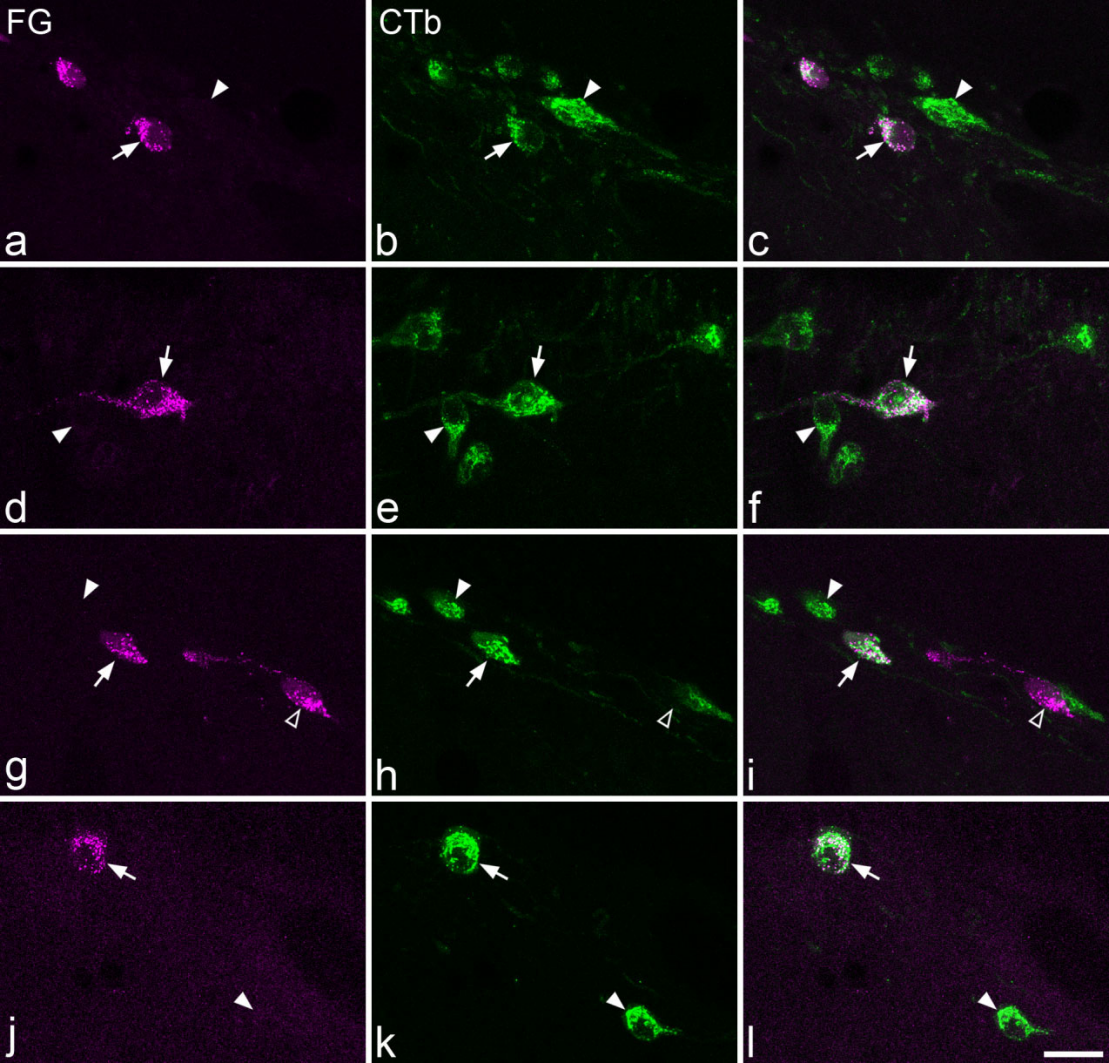
Retrograde labelling of lamina I neurons in transverse sections. In all cases Fluorogold (FG; transported from thalamus) is shown in magenta, and CTb (transported from LPb or PAG) is green. Arrows indicate double-labelled cells, solid arrowheads indicate cells labelled only with CTb, and the open arrowhead shows a cell labelled only with Fluorogold. **a**–**c**: Part of a section from C7 of experiment Pb1 contains several spinoparabrachial neurons, two of which are also labelled from the thalamus. **d**–**f**: Section from L4 of the same experiment shows spinoparabrachial neurons, one of which is labelled from thalamus. **g**–**i**: This field from C7 of experiment PAG3 contains single-labelled spinothalamic and spino-PAG neurons as well as a cell labelled from both sites. **j**–**l**: Section through L4 of the same experiment containing two spino-PAG cells, one of which is labelled from the thalamus. All images are obtained from 10 optical sections at 2-μm z-spacing. Scale bar = 20 μm.

In the Pb experiments, we found that all of the Fluorogold-positive (spinothalamic) lamina I neurons in C7 were also labelled with CTb and that the great majority (93%) of those in L4 were CTb labelled (Table 1). Double-labelled cells made up 45% of lamina I neurons labelled from LPb in C7 but only 6% of those in L4 (Table 1). In the PAG series, many of the lamina I spinothalamic cells (47%) in C7 were also labelled from the PAG, and these cells represented 58% of the spino-PAG population (Table 2). In the L4 segment, 33% of lamina I spinothalamic neurons were labelled from PAG (corresponding to 9% of spino-PAG cells; Table 2). Examples of single- and double-labelled lamina I neurons are illustrated in Figure 4.

**Figure 5:**
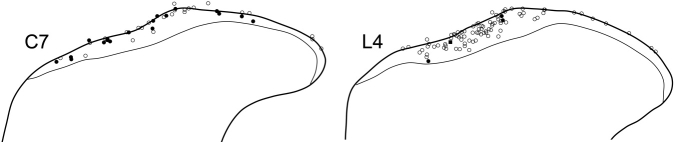
Plots of the locations of spinothalamic and spinoparabrachial neurons at cervical and lumbar levels. These drawings show the locations of all retrogradely labelled lamina I neurons in 10 alternate 60-μm sections through the C7 segment of experiment Pb2 and the L4 segment of Pb3. Open circles indicate cells labelled only from the LPb, solid circles are double-labelled cells, and the square is a cell that was labelled only from thalamus. The lower line indicates the approximate border between laminae I and II. There are more spinoparabrachial cells and many fewer spinothalamic cells in L4 compared with C7. Note that these drawings show 10 sections through the L4 segment (to allow direct comparison with C7), although the quantitative analysis of spinothalamic cells in L4 in these experiments presented in Table 3 was performed on 20 sections.

In experiments Pb1–3, retrogradely labelled cells positive for one or both tracers were present throughout the mediolateral extent of lamina I but were concentrated in its middle one-third. The numbers of spinothalamic cells and of cells labelled only from LPb that were present in the medial, middle, and lateral parts of lamina I in these three experiments are shown in Table 3 and examples of their distribution are illustrated in Figure 5. For each of these regions, there was no difference in the proportion of cells labelled from thalamus or only from LPb in either segment (χ^2^ test, *P* = 0.29 for C7, *P* = 0.18 for L4).

**TABLE 3:** Mediolateral Distribution of Spinothalamic and Spinoparabrachial Lamina I Neurons^1^

	C7			L4		
Projection target	Medial	Middle	Lateral	Medial	Middle	Lateral
Spinothalamic	5 (0–5)	34 (9–16)	22 (4–10)	7 (1–3)	16 (2–7)	8 (2–3)
Spinoparabrachial only	10 (3–4)	48 (11–22)	19 (6–7)	45 (13–17)	151 (40–63)	32 (7–16)

1 Total numbers of contralateral spinothalamic neurons and neurons labelled only from LPb (spinoparabrachial only) in medial, middle, or lateral one-thirds of lamina I in experiments Pb1-3. The ranges for each experiment are shown in parentheses. Spinothalamic neurons in L4 were counted from 20 sections, whereas all other groups were counted in 10 sections. See text for further details.

### Morphology, soma size, and NK1r expression of lamina I projection neurons in horizontal sections

This analysis was carried out on the C8 and L3 segments of experiments Pb1–3. In addition, for the morphological analysis, spinothalamic cells from the L5 segments of these experiments were added to the L3 sample. Altogether, 347 projection neurons were examined in the C8 segments (between 97 and 137 per experiment) and 239 neurons (53–98 per experiment) in L3/L5. Among these, 142 neurons (41–52 per experiment) in C8 and 55 neurons (16–20 per experiment) in L3/L5 were retrogradely labelled from the thalamus. All except one of the spinothalamic neurons in the lumbar segments and 140/142 of those in C8 were also labelled from LPb. When results from transverse and horizontal sections were combined, 201 of 203 (99%) spinothalamic neurons in C7–8 were labelled from LPb, and the corresponding value for L3–5 was 83 of 86 (97%). For each part of the subsequent analysis, data from the three experiments were pooled.

**Figure 6:**
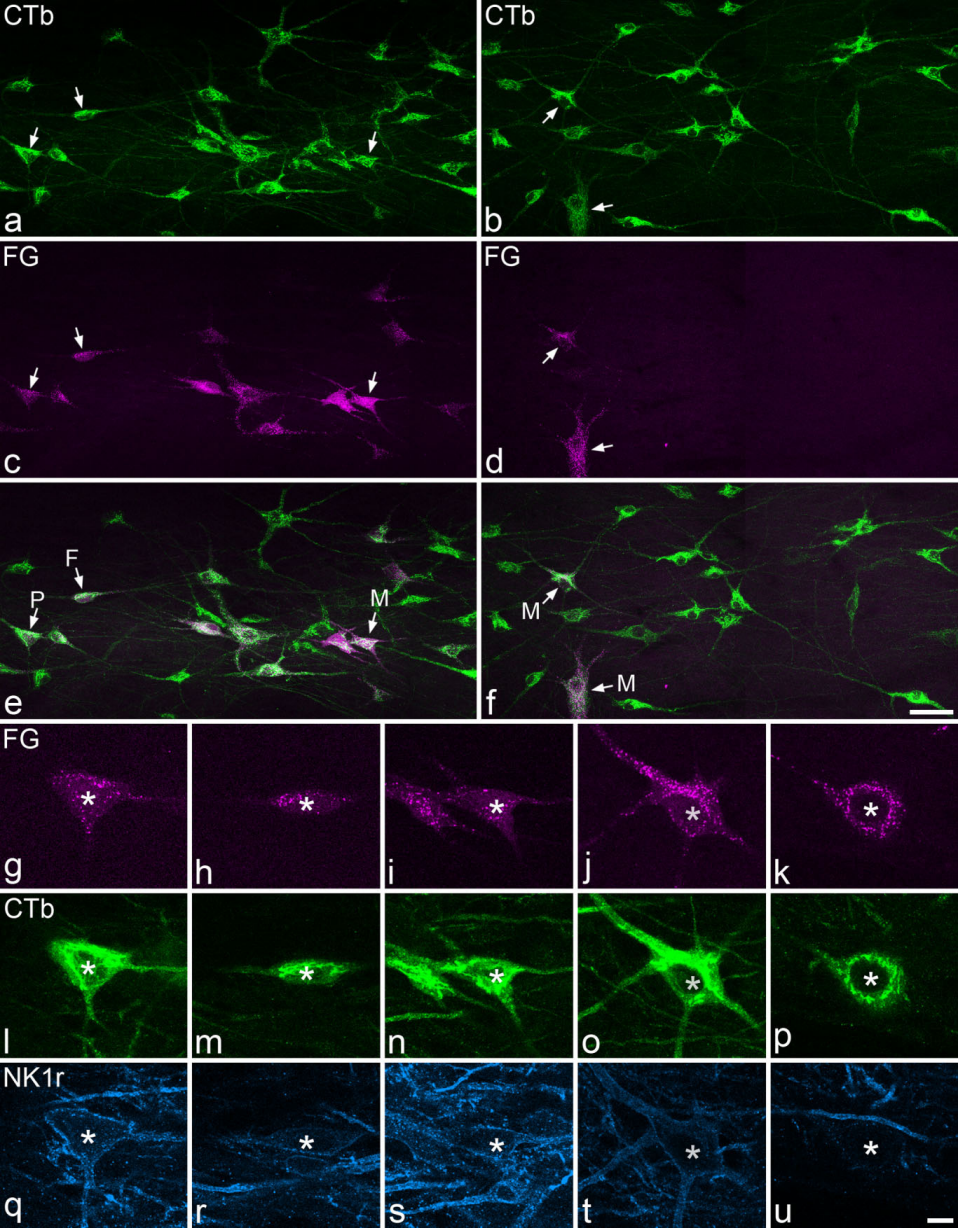
Lamina I spinoparabrachial and spinothalamic neurons in horizontal sections. **a**,**c**,**e**: Part of a section through the C8 segment of experiment Pb1. a: CTb (transported from LPb) is shown in green. c: Fluorogold (FG, transported from thalamus) is magenta. e: Merged image. Several spinoparabrachial neurons (three of which are indicated with arrows) are visible, and some of these are also retrogradely labelled from the thalamus. These cells have various shapes: those labelled P, F, and M are of pyramidal, fusiform, and multipolar types, respectively. **b**,**d**,**f**: Part of a section through L3 from Pb1 (colors as in a,c,e). Several spinoparabrachial neurons are visible, and two of these (arrows) are labelled from the thalamus. Both of these cells are multipolar (M). **g**–**u**: Higher magnification views through the five neurons marked with arrows in a–f, scanned to reveal Fluorogold (g–k), CTb (l–p), and NK1r (q–u). g,l,q: The pyramidal cell shows moderate (+++) NK1r immunoreactivity. h,m,r: The fusiform cell is very weakly immunoreactive (+) for NK1r. i,n,s: The multipolar cell was also classified as moderately (+++) NK1r-immunoreactive. j,o,t: The upper marked multipolar cell seen in f shows weak (++) NK1r immunostaining. k,p,u: The lower multipolar cell in f was classified as nonimmunoreactive (–) for NK1r. a,c,e and b,d,f are projections of 20 and 26 optical sections at 2-μm z-spacing, respectively. g–u are each projections of two optical sections at 1-μm z-spacing. Scale bars = 50 μm in f (applies to a–f); 10 μm in u (applies to g–u).

Although neurons belonging to each morphological type were seen within each projection population in both segments (Table 4), the proportions belonging to each type differed significantly between spinothalamic neurons and neurons labelled only from LPb (χ^2^ test, *P* < 0.05 for C8, *P* < 0.001 for L3/L5). Spinothalamic neurons were more often multipolar and less often fusiform than those labelled only from the LPb. This was particularly evident for the lumbar segments, where 32 of 55 (58%) spinothalamic neurons were classified as multipolar. Examples of neurons belonging to different morphological classes are illustrated in Figure 6.

**TABLE 4:** Morphology of Spinothalamic and Spinoparabrachial Lamina I Neurons^1^

	C8			L3/L5^2^		
Morphology	ST	SPb only	SPb total	ST	SPb only	SPb total
Multipolar	53 (37)	54 (26)	106 (31)	32 (58)	49 (27)	64 (31)
Pyramidal	35 (25)	42 (20)	77 (22)	12 (22)	50 (27)	55 (27)
Fusiform	35 (25)	74 (36)	108 (31)	9 (16)	70 (38)	71 (35)
Unclassified	19 (13)	35 (17)	54 (16)	2 (4)	15 (8)	15 (7)
Total	142	205	345	55	184	205

1 Numbers (and percentages) of contralateral lamina I neurons belonging to the spinothalamic (ST) or spinoparabrachial tracts that were assigned to each morphological class. Cells labelled only from LPb are shown as SPb only, whereas all of those labelled from LPb are shown as SPb total. Data were pooled from experiments Pb1-3. All of the spinothalamic neurons in L3, and all but two of those in C8, were also labelled from LPb and are therefore also classified as spinoparabrachial.

2 Note that the ST column includes data from both L3 and L5 segments, whereas the SPb only and SPb total columns contain data from the L3 segment only.

**Figure 7:**
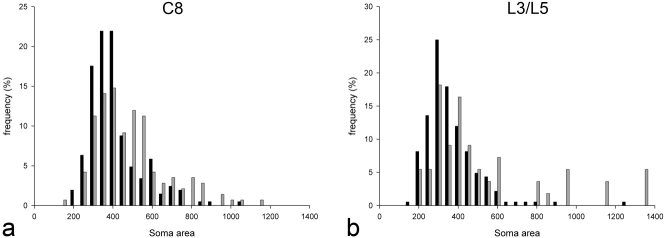
Soma areas of spinothalamic and other spinoparabrachial lamina I neurons. **a**: Histogram showing the distribution of cross-sectional areas of lamina I neurons labelled from the thalamus (gray bars) and of neurons that were labelled from LPb but not thalamus (black bars) in the C8 segment. **b**: Histogram showing the equivalent data for the lumbar enlargement. In this case, the sizes of spinothalamic neurons in both L3 and L5 segments are included (gray bars), whereas the sizes of neurons labelled from LPb but not thalamus (black bars) are from the L3 segment only.

The cross-sectional areas of cell bodies of spinothalamic neurons and those labelled only from LPb are shown in Figure 7. The soma areas of spinothalamic neurons ranged from 142 to 1,132 μm^2^ (median 434, n = 142) for those in C8 and from 173 to 1,350 μm^2^ (median 380, n = 55) for those in L3/L5, compared with 159–1,016 μm^2^ (median 354, n = 205) and 149–1,230 μm^2^ (median 313, n = 184) for neurons labelled only from LPb in C8 and L3, respectively. These differences were highly significant (Mann-Whitney rank sum test, *P* < 0.001 for both segments). Because we have recently reported that large gephyrin-coated lamina I neurons (which are either nonimmunoreactive or very weakly immunoreactive for the NK1r) make up ∼20% of the spinothalamic population in the L5 segment (Polgár et al., 2008), we also analyzed soma size for the cells that were assigned a strength of 2–4 for NK1r immunoreactivity in the C8 and L3 segments. For these cells, areas of spinothalamic neurons were 253–1,132 μm^2^ (median 477, n = 108) and 274–577 μm^2^ (median 398, n = 14) for C8 and L3, respectively. Corresponding values for spinoparabrachial neurons that were not labelled from thalamus were 241–1,016 μm^2^ (median 374, n = 137) for C8 and 163–1,230 μm^2^ (median 319, n = 115) for L3. The differences between the two projection populations were still highly significant (Mann-Whitney rank sum test, *P* < 0.005 for both segments).

Results of the analysis of NK1r expression are summarized in Table 5, and examples of immunostaining are shown in Figure 6q–u. NK1r immunoreactivity was detected on 82% and 81% of spinothalamic neurons in C8 and L3 and on 79% and 72% of spinoparabrachial neurons in these segments, respectively. Neurons with NK1r scores of 0 (negative) to 4 (strong) were found within each projection population in both segments. However, in C8, the strength of NK1r expression was significantly higher among spinothalamic neurons than among neurons labelled only from LPb (Mann-Whitney rank sum test, *P* < 0.05). Within this segment, NK1r immunoreactivity was scored 3 or 4 in 59% of spinothalamic neurons, but only in 43% of the other spinoparabrachial cells. There was no significant difference in NK1r strength among the two projection populations in L3 (Mann-Whitney rank sum test, *P* = 0.9). It has been reported that relatively few NK1r-immunoreactive spinothalamic cells are pyramidal (Yu et al.,^2005^), so we analyzed NK1r expression among pyramidal spinothalamic neurons in C8. We found that 76% of these cells were NK1r immunoreactive and that 64% of them were scored 3 or 4 for NK1r strength.

**TABLE 5:** Strength of NK1r Immunoreactivity in Spinothalamic and Spinoparabrachial Lamina I Neurons^1^

	C8			L3		
NK1r-ir strength	ST	SPb only	SPb total	ST	SPb only	SPb total
4	49 (35)	50 (24)	99 (29)	3 (14)	44 (24)	47 (23)
3	35 (25)	39 (19)	74 (21)	7 (33)	38 (21)	45 (22)
2	24 (17)	48 (23)	71 (21)	4 (19)	33 (18)	37 (18)
1	9 (6)	22 (11)	30 (9)	3 (14)	16 (9)	19 (9)
0	25 (18)	46 (22)	71 (21)	4 (19)	53 (29)	57 (28)
Total	142	205	345	21	184	205

1 Numbers (and percentages) of contralateral lamina I neurons belonging to the spinothalamic (ST) or spinoparabrachial (SPb) tracts that were assigned to different groups based on strength of NK1r immunoreactivity (NK1r-ir): 4 = strong, 3 = moderate, 2 = weak, 1 = very weak, 0 = negative. Data were pooled from experiments Pb1-3. Note that all of the spinothalamic neurons in L3, and all but two of those in C8, were also labelled from LPb and are therefore also classified as spinoparabrachial.

### Large gephyrin-coated lamina I neurons

These cells were identified in horizontal sections through the L5 and C6 segments. The results for L5 from experiments Pb1–3 and those from PAG1–2 for thalamic labelling of these cells in this segment have been reported previously (Polgár et al., 2008). In experiments PAG1–4, 32 large gephyrin-coated lamina I neurons were identified in the right dorsal horn in L5 (6–12 per experiment), and only two of these (6%) were retrogradely labelled from the PAG.

**Figure 8:**
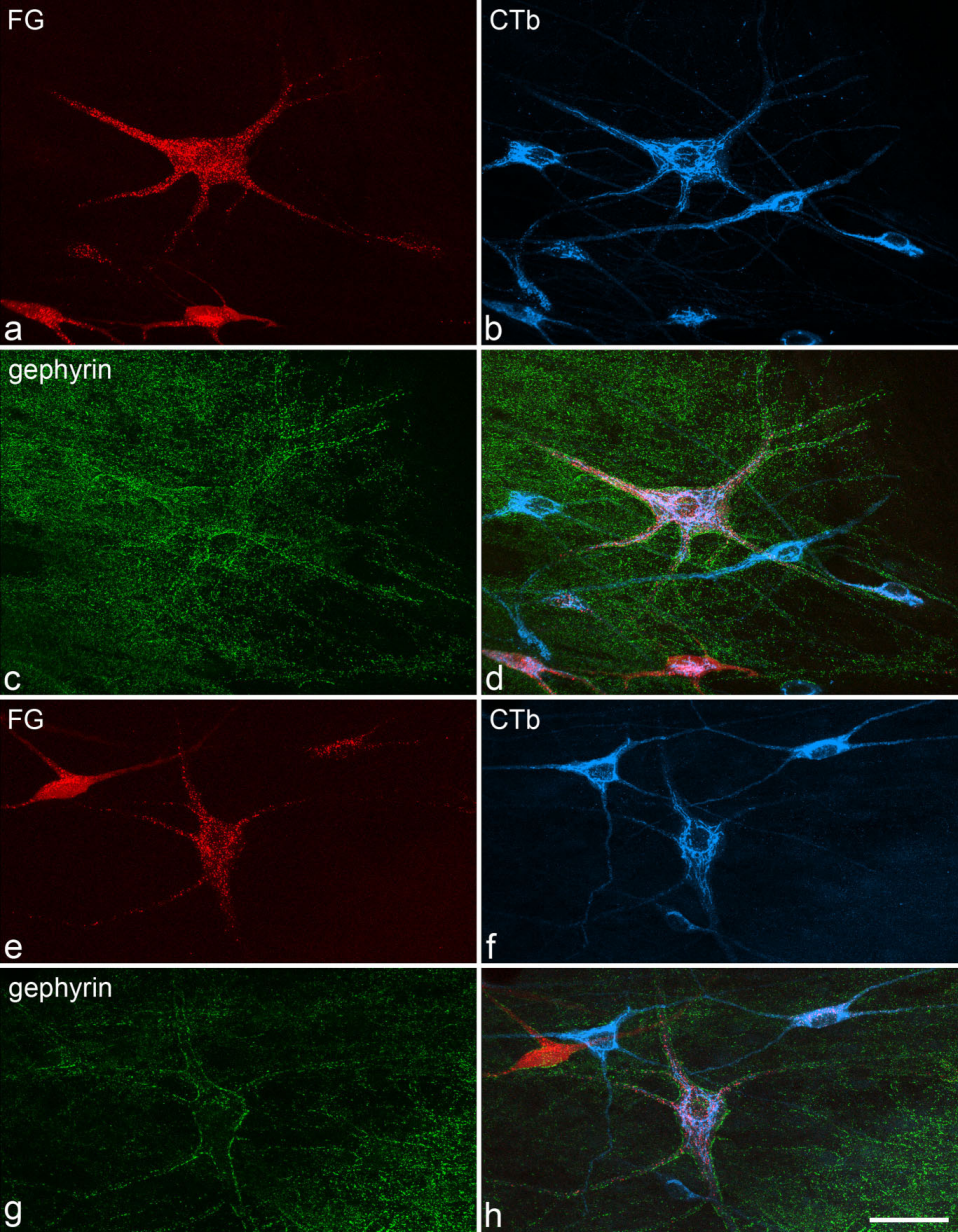
Large gephyrin-coated lamina I cells retrogradely labelled from thalamus, LPb, and PAG. Images from confocal scans through horizontal sections of C6 that show Fluorogold (FG; transported from thalamus, red), CTb (transported from LPb or PAG, blue), and gephyrin (green). **a**–**d**: This field from experiment Pb2 shows a large gephyrin-coated cell (center) that is retrogradely labelled from both thalamus and LPb. Several other CTb-labelled (spinoparabrachial) cells are also visible, and some of these also contain Fluorogold. **e**–**h**: Part of a section from experiment PAG4 shows a large gephyrin-coated cell (center) that is retrogradely labelled from thalamus and PAG. Other cells that are labelled from one or both of these regions are also visible. Images are projected from 24 (a–d) and 12 (e–h) optical sections at 2-μm z-spacing. Scale bar = 50 μm.

In the C6 segment, 33 gephyrin-coated cells were identified on the right side in sections from all seven experiments (two to six per experiment), and 27 of these (82%) were labelled with Fluorogold (i.e., were spinothalamic neurons). In the Pb experiments, 12 of 16 cells (75%) were CTb-labelled (spinoparabrachial), whereas, in the PAG experiments, 9 of 17 cells (53%) were CTb-labelled (spino-PAG). Most (11 of 16, 69%) of the cells in the LPb series and 7 of 17 (41%) of those in the PAG series were double-labelled. The mean number of gephyrin-coated cells identified in the C6 segments (4.7) was significantly lower than the mean number (8) found in the L5 segments in these experiments (*t*-test, *P* < 0.01). Examples of retrogradely labelled gephyrin-coated lamina I neurons in the C6 segment are shown in Figure 8.

### Lamina III/IV NK1r-immunoreactive projection neurons

The analysis of these cells was carried out on complete sets of serial transverse sections from the C7 and L4 segments, and results are shown in Tables 6 and 7. Because of variations in the lengths of spinal segments that were used from each experiment, the number of sections included in each series varied from 30 to 50 (corresponding to lengths of 1.8–3.0 mm). To allow a direct comparison between the numbers of cells of this type in the C7 and L4 segments, a correction was made by multiplying the observed total number of cells (Tables 6, 7) by the expected length of the segment (2.5 mm for L4, 2.3 mm for C7; Al-Khater et al., 2008) and dividing this by the actual length of the series (number of sections ×60 μm). This gave mean values of 15.9 cells for C7 and 22.9 cells for L4, and these were significantly different (*t*-test, *P* < 0.005). The cell bodies were located between 70 and 286 μm [mean 164 μm ± 47 (SD), n = 101 cells] below the dorsal white matter in C7 and between 100 and 367 μm (mean 239 μm ± 60, n = 170 cells) below the dorsal white matter in L4.

**TABLE 6:** Retrograde Labelling of Lamina III/IV NK1r Cells in Experiments Pb1-3^1^

	C7							L4						
Experiment	Total	FG only	CTb only	DL	Percent SPb	Percent ST	Percent DL	Total	FG only	CTb only	DL	Percent SPb	Percent ST	Percent DL
Pb1	12	0	1	11	100	91.7	91.7	26	4	10	3	50	26.9	11.5
Pb2	14	2	5	6	78.6	57.1	42.9	26	5	14	4	69.2	34.6	15.4
Pb3	12	2	3	7	83.3	75	58.3	19	2	11	3	73.7	26.3	15.8
Mean	12.7	1.3	3	8	87.3	74.6	64.3	23.7	3.7	11.7	3.3	63.8	29.3	14.2

1 Counts of large lamina III/IV NK1r-immunoreactive neurons identified in serial transverse sections through the C7 and L4 segments in these experiments. Cells were classified according to whether they contained only Fluorogold (FG only, from thalamus) or only CTb (CTb only, from LPb) or were double labelled (DL). The percentages of cells belonging to spinoparabrachial (SPb) or spinothalamic (ST) tracts and the percentage that were double labelled are also provided. Note that the length of the segment analyzed varied, so the total numbers do not exactly match those that would have been present in the entire segment (see text for further details).

**TABLE 7:** Retrograde Labelling of Lamina III/IV NK1r Cells in Experiments PAG1-4^1^

	C7							L4						
Experiment	Total	FG only	CTb only	DL	Percent SPAG	Percent ST	Percent DL	Total	FG only	CTb only	DL	Percent SPAG	Percent ST	Percent DL
PAG1	16	14	1	0	6.3	87.5	0	26	3	1	1	7.7	15.4	3.8
PAG2	14	9	0	2	14.3	78.6	14.3	27	6	1	2	11.1	29.6	7.4
PAG3	20	17	0	1	5	90	5	26	12	1	1	7.7	50	3.8
PAG4	13	13	0	0	0	100	0	20	3	1	0	5	15	0
Mean	15.8	13.3	0.3	0.8	6.4	89	4.8	24.8	6	1	1	7.9	27.5	3.8

1 Counts of large lamina III/IV NK1r-immunoreactive neurons identified in serial transverse sections through the C7 and L4 segments in these experiments. Cells were classified according to whether they contained only Fluorogold (FG only, from thalamus) or only CTb (CTb only, from PAG) or were double labelled (DL). The percentages of cells belonging to spino-PAG (SPAG) or spinothalamic (ST) tracts and the percentage that were double-labelled are also provided. Note that the length of the segment analyzed varied, so the total numbers do not exactly match those that would have been present in the entire segment (see text for further details).

**Figure 9:**
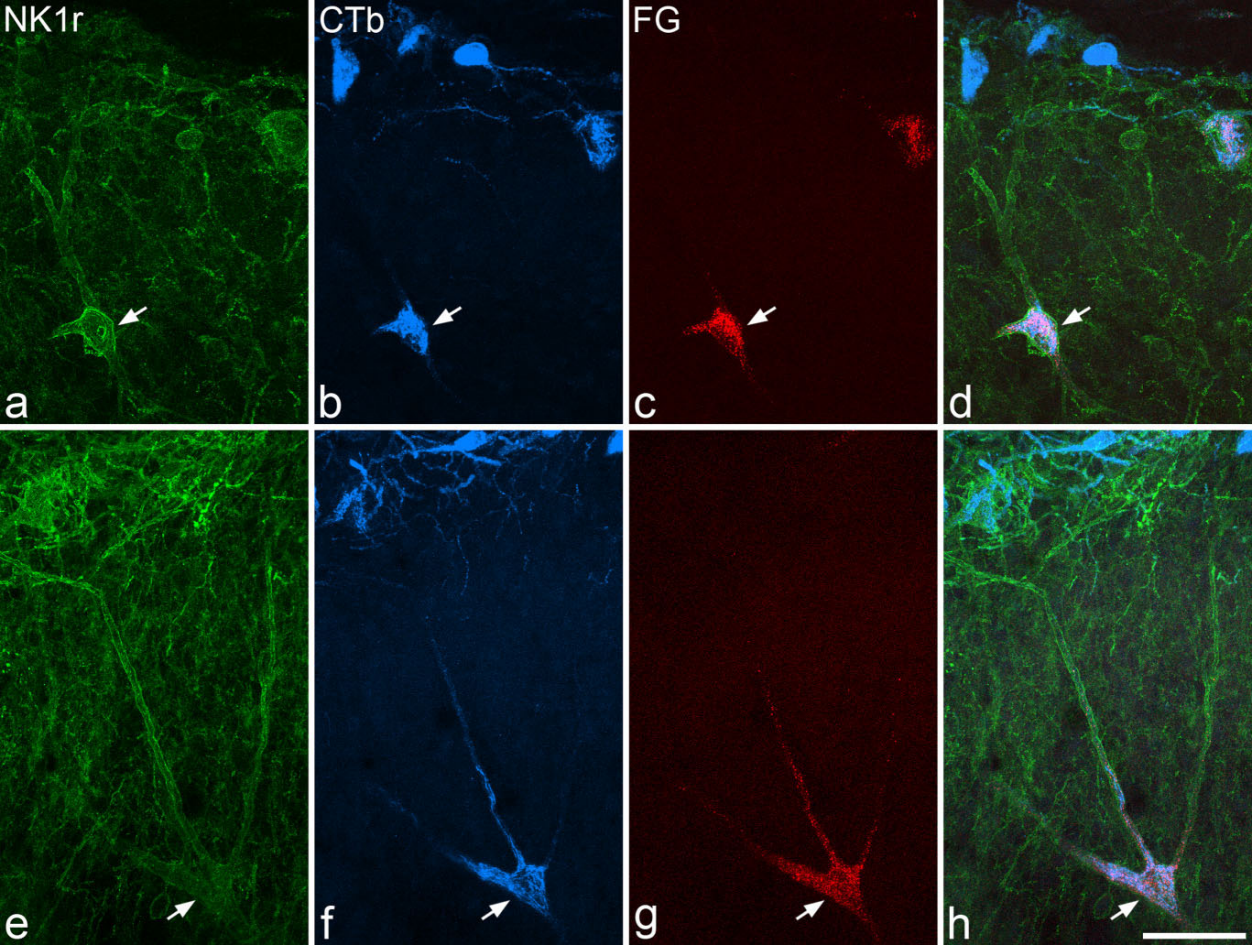
Retrograde labelling of lamina III/IV NK1r-immunoreactive neurons. These images are from confocal scans that show immunoreactivity for NK1r (green), CTb (transported from LPb, blue), and Fluorogold (FG; transported from thalamus, red) in transverse sections through C7 (**a**–**d**) and L4 (**e**–**h**) from experiment Pb3. In each case, a single large NK1r-immunoreactive cell with its soma in lamina III or IV is labelled with both retrograde tracers (arrows). Images are projections of 20 (a–d) or 18 (e–h) confocal optical sections at 2-μm z-spacing. Scale bar = 50 μm.

In the Pb experiments, most of the lamina III/IV NK1r cells in C7 were retrogradely labelled from thalamus (75%) or from LPb (87%), and 64% were labelled from both targets. In the L4 segment, 29% of these cells were labelled from thalamus and 64% from LPb, with 14% double-labelled (Table 6). Examples of double-labelled cells in both segments are shown in Figure 9. The proportion of these cells labelled from PAG was much lower in both C7 (6%) and L4 (8%), although the numbers labelled from thalamus were similar to those seen in the Pb series (89% and 28%, respectively, Table 7). In both segments, 4–5% of these cells were double-labelled. When results from all seven experiments were combined, the proportion of these cells labelled from thalamus was 83% for C7 and 28% for L4.

In the L4 segment, the proportion of lamina III/IV NK1r cells in the medial half of the dorsal horn retrogradely labelled from the thalamus was 35/72 (data pooled from all seven experiments), whereas the corresponding proportion for those in the lateral half was 14/98. These differed significantly (χ^2^ test, *P* < 0.001).

## DISCUSSION

The main findings of this study are that 1) the number of lamina I neurons retrogradely labelled from LPb is considerably lower in C7 than in L4, although numbers labelled from PAG in these segments are similar; 2) >95% of spinothalamic lamina I neurons in both enlargements are labelled from LPb, and between one-third and one-half are also labelled from PAG; 3) lamina I spinothalamic neurons differ from other neurons labelled from LPb in morphology, soma size, and strength of NK1r expression; and 4) many lamina III/IV NK1r-immunoreactive projection neurons in cervical enlargement and some of those in lumbar enlargement are labelled from both thalamus and LPb.

### Technical considerations

As in all retrograde tracing studies, it is necessary to consider the possibility of uptake of tracer by fibers passing near the injection sites. Axons from the spinal cord are located ventrally in the medulla (Lund and Webster, 1967; Mehler, 1979). In the pons, axons of lamina I spinoparabrachial neurons, which probably represent collateral branches (McMahon and Wall, 1985), ascend dorsally near the lateral aspect of the brainstem at a rostrocaudal level close to interaural 0, to enter the lateral parabrachial area. These give rise to a substantial plexus that occupies the lateral crescent, dorsal lateral, superior lateral, and external lateral nuclei of the LPb, together with a smaller projection to the Kölliker-Fuse nucleus (Slugg and Light, 1994; Bernard et al., 1995; Feil and Herbert, 1995). From here, axons travel dorsomedially into PAG and arborize extensively within its caudal part (Bernard et al., 1995; Feil and Herbert, 1995; Keay et al., 1997). Since injections of CTb into rostral PAG and surrounding regions label very few lamina I neurons (Lima and Coimbra, 1989), it is unlikely that spinothalamic axons reach the thalamus by passing rostrally from PAG. Anterograde tracing studies have not reported a rostral continuation of axons that have entered LPb from the superficial dorsal horn, apart from those that pass into the PAG, and it appears that the parent axons of lamina I spinothalamic tract neurons are located ∼500 μm lateral to the external lateral nucleus of the LPb (J.F. Bernard, unpublished observations).

Because the projection from superficial dorsal horn occupies most of the mediolateral extent of LPb and extends to its lateral edge, we aimed to fill the entire LPb with tracer in experiments Pb1–3. In Pb2, there was some extension of CTb lateral to LPb, and it is possible that in this case tracer was taken up by some spinothalamic axons. However, it is unlikely that this had a significant effect on the results, as CTb did not spread lateral to the LPb in the region occupied by these axons in experiments Pb1 and Pb3, and both the numbers of spinoparabrachial cells and the proportion of spinothalamic neurons labelled with CTb were consistent among the three experiments. The projection from superficial dorsal horn to PAG travels through the rostral part of the parabrachial area, and it is possible that axons of some lamina I cells project to PAG without arborizing in LPb. If this is the case, and if these cells were labelled through uptake of CTb into their axons within LPb, this would result in an overestimate of the proportion of spinothalamic neurons that projected to LPb. However, because many fewer cells are labelled from the PAG than from LPb, this is unlikely to have had a major effect on our results. In Pb1–3, there was some spread of CTb into other structures, such as the Kölliker-Fuse, medial parabrachial, and cuneiform nuclei. However, the Kölliker-Fuse nucleus is considered to be part of the parabrachial complex (Saper, 1995), while medial parabrachial and cuneiform nuclei apparently receive only a sparse input from the superficial dorsal horn (Bernard et al., 1995; Feil and Herbert, 1995).

It is very unlikely that CTb was taken up by spinothalamic axons in experiments PAG1–4, since these axons are located at a considerable distance lateral to the PAG. Although there was spread of tracer dorsally into the overlying part of the superior colliculus in PAG1 and PAG3, this is unlikely to have affected the results, as there is no evidence of a significant projection from superficial dorsal horn to this region (Beitz, 1982; Menétrey et al., 1982).

Gauriau and Bernard (2004a) reported that PoT is a major target for spinothalamic axons originating from superficial dorsal horn in the rat, and we subsequently demonstrated that most (if not all) of the lamina I component of the tract, as well as the part originating from NK1r-immunoreactive lamina III/IV neurons, could be retrogradely labelled from tracer injections into this region (Al-Khater et al., 2008). Although there was some spread of Fluorogold into regions outside the thalamus, these are not thought to be a major target for axons of superficial dorsal horn neurons (Gauriau and Bernard, 2004a).

### Differences in lamina I projections between cervical and lumbar enlargements

The results of the present study suggest that, compared with L4, C7 contains fewer spinoparabrachial cells, a similar number of spino-PAG cells, and many more spinothalamic cells in lamina I. The number of lamina I spinothalamic neurons has been found to be higher in cervical than in lumbar enlargements in rat, cat, and monkey (Kevetter and Willis, 1983; Harmann et al., 1988; Burstein et al., 1990a; Dado and Giesler, 1990; Zhang et al., 1996; Zhang and Craig, 1997; Kobayashi, 1998; Klop et al., 2005; Al-Khater et al., 2008). In part, this presumably reflects the greater representation of forelimb compared with hindlimb in somatosensory cortex (Kaas, 1983; Remple et al., 2003). However, the difference appears to be considerably greater in the rat, since Zhang and Craig (1997) found approximately twice as many lamina I spinothalamic cells in C6–8 as in L5–7 in the monkey, and the present results indicate that the difference between C7 and L4 in rat is more than fourfold. The difference between cervical and lumbar enlargements may be less dramatic for neurons that project to the ventral posterolateral (VPL) nucleus and thus convey information to primary somatosensory (S1) cortex, since some lamina I spinothalamic neurons in rat cervical enlargement project only to PoT or surrounding regions (Zhang and Giesler, 2005), while it is not known whether this is the case for those in the lumbar enlargement. Zhang et al. (2006) reported that cervical lamina I neurons projecting to VPL often had large receptive fields, covering several digits and extending proximally on the limb. If this is the case for lumbar cells, it may explain why so few are needed to provide input to S1 cortex from the entire dermatome. Nonetheless, the larger number of spinothalamic lamina I cells in cervical segments presumably results in more accurate stimulus localisation in forelimb compared with hindlimb. Nociceptive information from the hindlimb is also thought to reach the brain through short-fiber multisynaptic pathways (Basbaum, 1973), and these presumably supplement direct pathways, such as the spinothalamic tract.

It is unlikely that we failed to label a significant number of spinoparabrachial cells in C7, because projections from superficial dorsal horn of lumbar and cervical enlargements terminate in similar areas of LPb (Slugg and Light, 1994; Bernard et al., 1995; Feil and Herbert, 1995; Saper, 1995), which were included in our injection sites. In addition, we found that virtually all lamina I spinothalamic neurons in C7 were labelled from LPb. If we had missed a significant number of spinoparabrachial cells, we would have expected a much larger number of cells retrogradely labelled only from thalamus. The lower number of spinoparabrachial cells in C7 is not due to a difference in the size of lamina I, as the mediolateral extent of the dorsal horn is similar in C7 and L4. The difference may reflect relative sizes of dermatomes, because the L4 dermatome is considerably larger than that of C7 (Takahashi and Nakajima, 1996). The main outputs from regions of LPb innervated by lamina I neurons are the amygdala and hypothalamus, which are thought to play a role in affective and autonomic aspects of pain (Bernard et al., 1996). Although the major targets of PoT are secondary somatosensory (S2) and insular cortices, PoT also projects to the amygdala (Ottersen and Ben Ari, 1979; Gauriau and Bernard, 2004b), which will presumably receive a much smaller input through the spinothalamic tract from the lumbar enlargement. In addition, the number of spinohypothalamic lamina I neurons is considerably lower in lumbar than in cervical segments (Burstein et al., 1990b). The larger number of spinoparabrachial cells in lumbar cord may therefore compensate for the reduced input that this region provides to both amygdala and hypothalamus through spinothalamic and spinohypothalamic tracts. The similarity in numbers of spino-PAG cells in C7 and L4 presumably reflects an equivalent importance of the two segments in activating antinociceptive and other coping mechanisms.

We have previously provided evidence that injection of tracer into LPb labels ∼85% of all lamina I projection neurons in L4 of the rat and that these include >95% of those that project to PAG (Spike et al., 2003). The lower number of cells labelled from LPb in C7 therefore suggests that there are many fewer lamina I projection neurons per segment in cervical enlargement. Consistently with this, we have found that virtually all spino-PAG neurons in C7 are also labelled from LPb (A.J. Todd, unpublished observations). Although, this cannot be confirmed until numbers and collateral projections of cervical lamina I cells labelled from other targets (e.g., caudal ventrolateral medulla and nucleus of solitary tract) have been determined, the present results suggest that there is a dramatic difference between the two enlargements in the proportion of lamina I projection neurons that belong to the spinothalamic tract.

### Collateral projections of spinothalamic neurons

The proportions of spinothalamic cells that were also labelled from LPb in the present study were somewhat higher than those reported by Hylden et al. (1989), presumably because they injected fluorescent beads (which give very restricted injection sites) into LPb and therefore did not label all spinoparabrachial cells. However, Hylden et al. reported that 31% of lumbar spinoparabrachial lamina I neurons projected to thalamus, and this is difficult to explain in light of previous estimates of the numbers of spinothalamic and spinoparabrachial lamina I neurons in the lumbar enlargement (see above). Our finding that relatively few (6%) spinoparabrachial cells in this region project to thalamus is consistent with the report by McMahon and Wall (1985), who examined axons of 13 lamina I projection neurons in rat lumbar enlargement and found that all of them gave collaterals to midbrain (including PAG and an area that corresponds to LPb), whereas none could be activated from more rostral areas.

Previous studies in rat have indicated that some lamina I cells project to both thalamus and PAG. Liu (1986) observed a few double-labelled neurons in lamina I but did not state the proportion of labelled cells that contained both tracers. Harmann et al. (1988) reported that lamina I neurons labelled from both sites made up only ∼10% of spino-PAG and <5% of spinothalamic neurons. In the present study, we found a much higher degree of double labelling in C7 (58% of spino-PAG cells and 47% of spinothalamic cells) and a higher proportion of spinothalamic cells labelled from PAG (33%) in L4. These discrepancies are probably due to differences in the injection sites.

Our finding that virtually all spinothalamic lamina I neurons were labelled from LPb and that a significant proportion were labelled from PAG suggests that nearly half of the spinothalamic lamina I cells in C7 and almost one-third of those in L4 send collateral branches to both LPb and PAG. This indicates that individual lamina I neurons may contribute to several different functions, including stimulus localization and affective aspects of pain (through projections to thalamus and LPb) as well as activation of descending modulatory influences (through projections to PAG).

We previously reported that 85% of large lamina III/IV NK1r-immunoreactive cells in C6, and 17% of those in L5, were spinothalamic, and these appear to project to PoT but not to VPL (Al-Khater et al., 2008). Our present finding for C7 is very similar to that for C6, although the percentage for L4 (28%) is somewhat higher than we had observed in L5. Most of those in C7 were labelled from both thalamus and LPb, whereas, in L4, approximately half of those projecting to thalamus were labelled from LPb. Our results for both C7 and L4 indicate that PAG is not a major target for axons of these cells. As in our previous study, we found that the proportion belonging to the spinothalamic tract in lumbar enlargement was significantly higher for those in the medial part of the dorsal horn, which receives inputs from the distal part of the hindlimb, and this may reflect the relative importance of distal hindlimb input to S2 cortex.

### Differences between projection populations in lamina I

Our results indicate that spinothalamic lamina I cells differed from other neurons labelled from LPb in terms of morphology, soma size, and (in the C8 segment) strength of NK1r expression. Several studies have examined morphology of lamina I spinothalamic cells, but these have not revealed a consistent pattern. For rat, Lima and Coimbra (1988) reported that these cells belonged to pyramidal or flattened (multipolar) classes, whereas Yu et al. (2005) found that fusiform cells were most numerous, particularly in the cervical enlargement. Consistently with our previous study (Spike et al., 2003), we found that the proportion of spinoparabrachial lamina I cells in L3 that belonged to each morphological class was similar, and a comparable result was found in C8. However, spinothalamic lamina I cells differed significantly from other neurons labelled from LPb in that more were multipolar, particularly in the lumbar region, where multipolar cells made up 58% of the population. One reason for the high proportion of multipolar spinothalamic cells in lumbar enlargement is that large gephyrin-coated cells (most of which are multipolar) make up ∼20% of the lamina I spinothalamic population at this level (Polgár et al., 2008). These are also present in the cervical enlargement but are greatly outnumbered by other spinothalamic cells and cannot therefore account for the relatively high proportion of multipolar cells in this region. Spinothalamic lamina I cells also had significantly larger somata than other cells labelled from LPb. Again, this is due in part to the presence of large gephyrin-coated neurons within this population, but, when we excluded cells that were negative or very weak for NK1r (which should account for all of the gephyrin-coated cells), we still found a significant difference in soma size. Electrophysiological studies in the rat suggest that spinothalamic lamina I neurons generally have larger receptive fields than those of spinoparabrachial neurons (Bester et al., 2000; Zhang and Giesler, 2005; Zhang et al., 2006). Bester et al. (2000), who analyzed 53 spinoparabrachial neurons in lumbar enlargement, reported that these had receptive fields restricted to one or two digits, and the present results suggest that few of these would have belonged to the spinothalamic tract. Because primary afferent input to the dorsal horn is somatotopically organized, receptive field size of lamina I neurons is probably correlated with the extent of their dendritic trees. It is therefore likely that the difference in cell body size is at least partially accounted for by the need for spinothalamic neurons to support larger dendritic trees. In C8, the strength of NK1r immunoreactivity was significantly higher among spinothalamic neurons, and we have previously observed that lumbar lamina I cells with strong NK1r immunoreactivity are under-represented among the population that projects to PAG (Spike et al., 2003). Taken together, these results suggest that subpopulations of lamina I cells differ in their pattern of supraspinal projection.

Han et al. (1998) reported that there was a close relationship between morphology and function for lamina I neurons in cat spinal cord, with pyramidal cells responding only to innocuous cooling and those in the other two morphological classes being activated by noxious stimuli. Consistently with this suggestion, Yu et al. (2005) and Almarestani et al. (2007) reported that pyramidal cells projecting to thalamus or LPb in the rat were seldom NK1r immunoreactive. However, although cooling-sensitive lamina I spinothalamic neurons have been identified in the rat, these cells also responded to noxious mechanical stimuli (Zhang et al., 2006). If these are included among the pyramidal class, they may correspond to some of the NK1r-positive pyramidal spinothalamic neurons seen in the present study. However, it is unlikely that all pyramidal lamina I neurons in the rat respond to innocuous cooling, as 23–30% of lumbar spinoparabrachial cells are pyramidal (Spike et al., 2003; Almarestani et al., 2007; present study), and it has been reported that lamina I spinoparabrachial neurons are not activated by innocuous cooling (Bester et al., 2000). Nonetheless, we have observed functional differences related to morphology in lamina I of rat spinal cord; NK1r-positive multipolar projection neurons were more likely to express Fos in response to noxious cold stimuli than those belonging to other morphological classes (Todd et al., 2005). However, we have also found differences among multipolar lamina I cells: although the great majority of both gephyrin-coated and NK1r-expressing multipolar projection neurons up-regulated Fos following subcutaneous formalin injection (Puskár et al., 2001; Todd et al., 2002), only 38% of the gephyrin-coated cells did so in response to a noxious heat stimulus that caused Fos expression in 85% of NK1r-immunoreactive multipolar cells (Polgár et al., 2008). Although most, if not all, lamina I projection neurons in the rat respond to noxious stimuli, there may be differences in the types of information transmitted to different supraspinal targets by subpopulations of these cells.
